# Molecular detection and phylogeny analysis of *Coxiella burnetii* detected from cattle and buffalo milk based on plasmid *cbhE* gene in West Azerbaijan of Iran

**DOI:** 10.1016/j.nmni.2024.101495

**Published:** 2024-10-11

**Authors:** Peyman Khademi, Amir Tukmechi, Abdulghaffar Ownagh

**Affiliations:** a Postdoc, Department of Microbiology, Faculty of Veterinary Medicine, Urmia University, Daneshgah Blvd, Urmia, West Azerbaijan, Iran; b Department of Microbiology, Faculty of Veterinary Medicine, Urmia University, Daneshgah Blvd, Urmia, West Azerbaijan, Iran

**Keywords:** Buffalo, Cattle, *Coxiella burnetii*, Milk, Nested-PCR, Plasmid

## Abstract

Humans and animals may get Q fever, which is caused by the Gram-negative coccobacillus *Coxiella burnetii*. The symptoms of Q fever may include a self-limiting febrile illness, pneumonia, endocarditis, or hepatitis. Infections are classified as either acute or persistent. Cattle, sheep, and goats are the most prevalent reservoir animals for this zoonosis. This research was conducted to identify *C. burnetii* using transposable and isocitrate dehydrogenase genes (*IS1111*, *icd*) and *QpH1* plasmids. A total of 142 samples of raw buffalo and cow milk were collected from various locations within the West Azerbaijan region (see map). We used "nested" PCR techniques using primers based on the *IS1111* and *icd* genes of *C. burnetii*, as well as conserved and variable portions of plasmid sequences, to identify *C. burnetii* and their plasmids in milk samples from buffalo and calves. Out of 142 milk samples that were positive for the chromosomal transposable genes (*IS1111* and *icd*) at a rate of 16.9 percent (95 percent CI: 14.5 percent to 19.6 percent) and 7.1 % (95 percent CI: 5.59 percent to 9.08 percent), respectively, 86 samples were positive for the *QpH1* plasmid at a rate of 60.5 percent (95 percent CI: 52.35 percent to 68.2 percent). Based on a phylogenetic study of the *icd* and *QpH1* genes, the majority of the isolates had a similarity of 99.45–99.9 percent. Conclusion: It was determined that the buffalo population in West Azerbaijan province represents a significant epidemiological factor with respect to Q fever and consequently public health.

## 1 Introduction

Q fever is a zoonotic disease that caused by *Coxiella burnetii*, a Gram-negative bacterium that affects humans and animals. Infections can be transmitted to humans through inhalation of bacteria from infected animals, direct contact with infected animals, or consumption of contaminated products such as milk and dairy items [1,2].

Q fever in humans is mostly transmitted by domestic animals such as sheep, goats, and cattle [3]. *C. burnetii* is shed in milk, urine, feces and birthing materials [4]. Q fever is an underreported and poorly understood illness that affects people throughout the year. However, the majority of instances occur in the spring and early summer, particularly during calving and lambing season. *C. burnetii* is found in large amounts in birthing materials from infected animals whether they exhibit symptoms or not. While large amounts of bacteria can sometimes be found in milk, it is not present in urine and feces [4].

However, lipopolysaccharide, restriction fragment length polymorphism [5], and plasmid types are utilized to distinguish between acute and chronic *C. burnetii* isolates [5]. The techniques used to determine the type of *C. burnetii* strains include Genome RFLP, PFGE, Sequencing, and PCR-RFLP. More recently, two PCR-based techniques, MLVA and MST, have been developed to specify *C. burnetii* types without the need for bacterial isolation (OIE). These genotyping methods are essential in distinguishing between strains of the same species, which has significant implications for disease diagnosis, treatment, outbreak investigation, surveying epidemics, and conducting epidemiological studies. Whole genome sequencing is the fundamental technique used to compare different strains. Fortunately, advancements in sequencing technologies have resulted in a considerable reduction in costs. However, it may still present challenges for many laboratories when it comes to strain comparison [6].

Several important genotyping methods have been employed to study *C. burnetii*, including com1, *IS1111* Sequencing, SNP, RFLP, MLST, and MLVA [6]. It's worth noting that acute isolates of *C. burnetii* possess a 36-kbp plasmid called *QpH1*. DNA hybridization studies have revealed that *QpH1* contains a 6-kbp region of DNA that is not found in the *QpRS* plasmid from chronic isolates [7].

When epidemics occur, Q fever significantly impacts public health. Each epidemic typically involves up to 415 laboratory-confirmed human cases and these cases are regularly reported globally. Outbreaks of Q fever is usually confined to specific areas and are short-lived, lasting only for one episode. Sheep are the primary source of these outbreaks, with goats being the secondary source. However, the Dutch Q fever epidemic was exceptional due to its duration. Additionally, there have been documented instances of outbreaks associated with the consumption of cow and buffalo (buffalo) milk [8].

Thus, genotyping of the *C. burnetii* strains implicated is preferred for source identification and confirmation of the connection to human illness. In the investigation by Porten et al. (2006), nine isolates were grouped together with sheep and a human isolate was connected to a Q-fever epidemic that occurred in North Rhine Westphalia in 2003 [9]. Earlier research [10] indicates that Q fever is a problem for public health in several locations in Iran. Additionally, in the study by Mobarez et al. (2022), five genotypes of *C. burnetii* were identified among ruminants in Iran using the MST method [11]. Data on the prevalence of Q fever in domestic and wild animals also suggest that Q fever is common in Iran. It has been reported that a lack of level 3 diagnostics laboratory infrastructure and a lack of knowledge may make it difficult to quickly identify cases of Q fever, thus endangering public health. The prevalence of *C. burnetii* in milk from different regions of Iran has been reported to range from 4.22 % to 25.55 % [12]. Urmia city is the center of West Azerbaijan province, located in the northwest of Iran. Iran has approximately 523 thousand buffaloes, 27.7 % of which are Azeri buffaloes, residing in West Azerbaijan province [13]. In this research, our objective was to identify *C. burnetii* by employing primers designed for *IS1111* and *icd* genes, while also verifying the presence of the *QpH1* plasmid to distinguish between acute and chronic forms of the disease.

## 2 Material and methods

### 2.1 Stady area

**Fig. 1:**
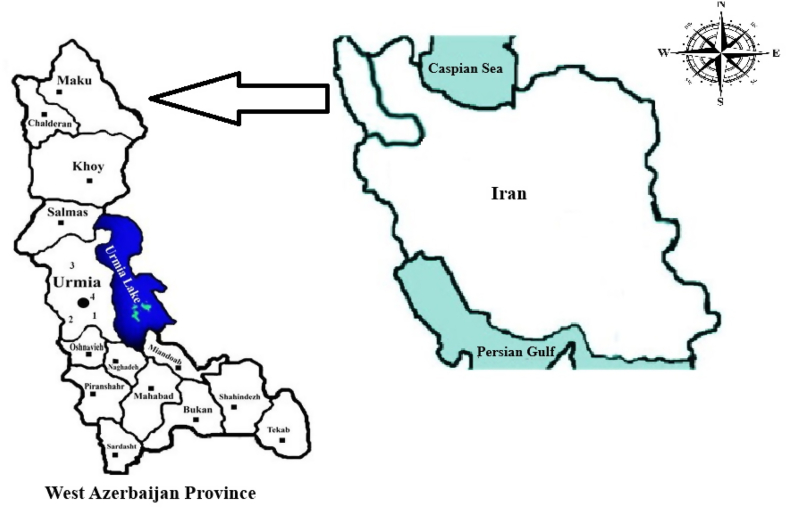
The schematic map of the study areas, West Azerbaijan province, Iran.

The present study was conducted in West Azerbaijan Province, situated in the northwestern region of Iran. Its geographical coordinates are 37° 33′ 10.08″ N, 45° 4′ 33.24″ E (https://tools.wmflabs.org). The climate of West Azerbaijan is significantly influenced by the moist winds originating from the Atlantic Ocean and the Mediterranean. The province is subject to the influence of cold northern winds during the winter months, resulting in the accumulation of significant snowfall (https://www.britannica.com/place/Azerbaijan-region-Iran) (Fig. 1).

### 2.2 Milk sampling

In this investigation, 840 milk samples from 420 cattle and 420 buffaloes from 84 dairy farms were collected over the course of four seasons in 2021 in the north, center, and south of West Azerbaijan province. Three age categories of the sampled animals <6 years, 6–10 years, and older than 10 years were created. All samples were sent in a cold bag to the Microbiology Laboratory.

### 2.3 DNA extraction from milk samples

In order to extract DNA from milk samples, the steps outlined by Berri et al. were followed. In brief, milk samples were centrifuged to produce pellets [14]. The pellets were periodically washed with sterile PBS. The sample precipitate was mixed with 200 μL of PBS buffer from Merck, Germany. Then it was centrifuged at 13000 rpm. DNA was extracted using a DNA extraction kit (Favorgen, Taiwan). Thermo Scientific's Nanodrop 2000c was utilized for DNA quality checking. To be used in PCR later, extracted DNA samples were stored at −20 °C.

### 2.4 Primers

All primers applied in present research are summarized in (Table 1). For molecular detection of *C. burnetii* nested-PCR targeting the transposon *IS1111* and isocitrate dehydrogenase *icd* genes was used. Primer sets of CB5-CB6 and QpH1F-QpH1R were used to target a specific gene of the *QpH1* plasmid, *cbhE* [5,15]. For the second PCR step, the primers QpH1R and QpH1F were designed using the Amplifx software (version 07, 0, 2; France). The specificity of the designed primers was verified using the Blast program on the NCBI website.

**Table 1:** The amplification protocol names, thermal program for both touchdown and Trans PCR and primer names and sequences and the size of PCR products [5,15].

Protocol	Primer Name	Sequence 5'----3'	PCR product size (bp)	PCR condition (Cycle)	Reference
Trans-PCR	IS-F	CAGAGCCACCGTATGAATCAGCTT	960	95c for 3m, 94 c for 30s, 62–66 (5) for 30s, 72c for 1m, 72c for 10m. (35)	This study
	IS-R	TCGGACGTTTATGGGGATGGGTAT			
nested-PCR	IS-NF	CACATTGCCGCGTTTACTAATCCC	421	95c for 3m, 94 c for 30s, 54 for 20s, 72c for 1m, 72c for 10m. (35)	
	IS-NR	CACGGCGCTGATCAATGAGATTC			
PCR	icd-F	CGGAGTTAACCGGAGTATCCA	738	Touchdown PCR (94 for3m, 94 for2m, 65-55 for1m, 77 for75s, 77 for5m) (3) 93 for4m, 94 for20s, 55 for1m, 77 for75s, 77 for5m. (30)	This study
	icd-R	CCGTGAATTTCATGATGTTACCTTT			
nested-PCR	icd-NF	GATATCGCGCCCGTCATGAAAA	556	94 for2m, 94 for30s, 55 for20s, 75 for75s, 75 for5m. (35)	
	icd-NR	CGGAATCGCGGTCATTATCAATGG			
PCR	CB5	ATAATGAGATTAGAACAACCAAGA	977	94 for 4m, 94 for2m, 53 for1m, 72 for2m, 72/5. (35)	(Zhang et al., 1998)
	CB6	TCTTTCTTGTTCATTTTCTGAGTC			
nested-PCR	QpH1-F	CTCGCTGACGGAAGAGGATCTTTT	602	94 for3m, 94 for45s, 50 for45s, 72 for45s, 72 for5m. (35)	This study
	QpH1-R	TAACACTGCCCGTCGCTTTACT			

### 2.5 Nested-PCR for the molecular detection of C. burnetii

Using Taq DNA Polymerase Master Mix RED, the *IS1111* and *icd* genes were the targets of the first step of the nested-PCR (Amplicon, Denmark). The 25 μl reaction volume used for the PCR reaction included 12.5 μl of mater mix, 50 pmol of each primer (ISNF & ISNR and icd-F & icd-F), and 5 μl of extracted DNA. The touchdown PCR was employed to improve the reaction's specificity and sensitivity while lowering contamination and inhibitor levels. The thermal cycler (Quanta Biotech, England) was used to define the touchdown PCR thermal protocols (Table 1) as previously mentioned. Overall, the nested-PCR stage is given with 2.5 μl of the 50:50 diluted PCR product, it should be mentioned. During DNA extraction procedure, elution buffer from the extraction kit was used as Negative Control of Extraction. Positive Control; The *C. burnetii* isolates used in this study comprised one strain (*C. burnetii* standard Nine Mile strain RSA 493). Negative control; In this study, in all stages of PCR, a negative control was used, which included master mix, distilled water, and primer.

### 2.6 Amplification of QpH1

Two sets of modified primers were used to identify the specific plasmid sequence of *C. burnetii*. The first set of primers, CB6-CB5, was designed to identify the *cbhE* gene of the QpH1 plasmid. Using nested PCR, the conserved region of plasmids (QpH1) was amplified. 20 μl reaction volume used for the PCR reaction included 12.5 μl of Taq DNA Polymerase Master Mix RED, 50 pmol of each primer, and 5 μl of extracted DNA (Amplicon, Denmark). Used was the touchdown PCR (Table 1). For the nested-PCR step (thermal cycling condition in (Table 1), 3 μl of the first PCR product that had been diluted (1:9) was utilized.

### 2.7 Electrophoresis

All PCR products were separated in a 2–2.5 % agarose gel and the gel photographed applying ultraviolet trans illumination (Syngene Bio-Imaging, UK).

### 2.8 Sanger sequencing

Four PCR products from samples, together with the amplified *cbhE*′ gene fragment (602 bp) for seven samples, were delivered to SinaClon Company in Tehran, Iran, for sequencing. Using the sophisticated BLAST similarity search tool, obtained nucleotide sequences were checked against GenBank (National Center for Biotechnology Information, Rockville Pike, and Bethesda, USA) and compared to the identical sequences of *C. burnetii* isolates from GenBank. Clustal W was used to align and compare nucleotide sequences to other nucleotide sequences from GenBank, and MEGA software version X was used to create the phylogenetic tree using the neighbor-joining method.

### 2.9 Statistical analysis

Data were analyzed applying the Chi-square test in SPSS version 22 (IBM Corp. Armonk, NY, USA). Differences with a p-value <0.05 were regarded significant.

## 3 Results

### 3.1 Detection of C. burnetii in raw milk of buffalo and cattle

**Fig. 2:**
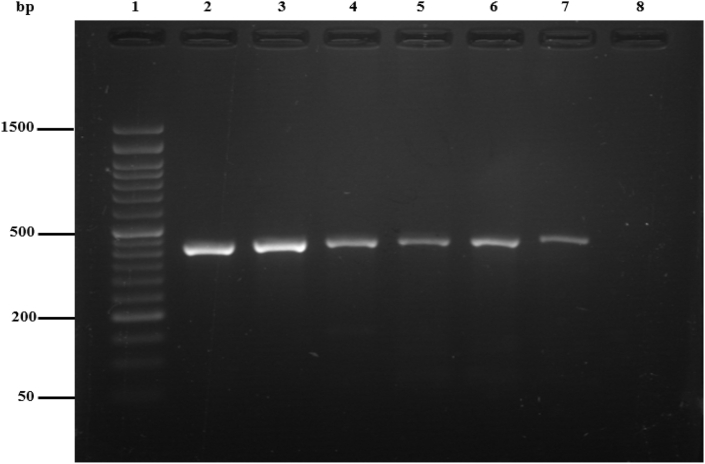
Agarose gel image of amplified fragment of *C. burnetii IS1111* gene (421bp) using nested-PCR. Lane 1, 50-bp molecular ladder (Smobio Technology Inc., Taiwan); lane 2 positive control sample, lanes 3, 4, 5, 6 and 7 positive samples for *C. burnetii*, lane 8, negative control. Positive control (*C. burnetii* standard Nine Mile strain RSA 493 Nine).

**Fig. 3:**
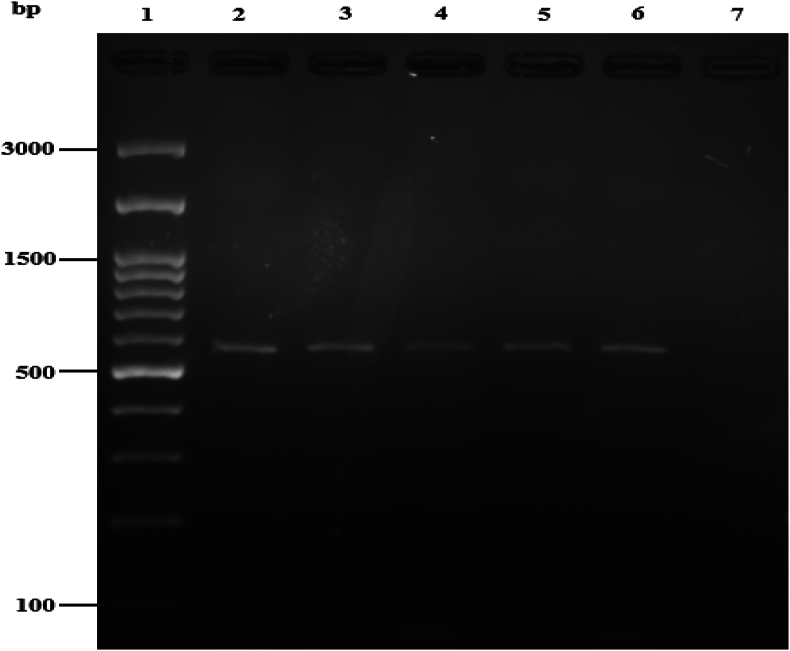
Agarose gel image of amplified fragment of *C. burnetii icd* gene (556bp) using nested-PCR. Lane 1, 100-bp molecular ladder (Smobio Technology Inc., Taiwan); lane 2 positive control sample, lanes 3, 4, 5 and 6 positive samples for *C. burnetii*, lane 7, negative control. Positive control (*C. burnetii* standard Nine Mile strain RSA 493 Nine).

Out of all the milk samples that were examined, 142 samples tested positive, accounting for 16.9 % of the total. Specifically, 81 (19.3 %) of the positive samples were derived from buffalo milk (11 samples for <6, 59 samples for 6–10 and 11 samples for >10 age group), while 61 (14.5 %) were from cattle (9 samples for < 6, 44 samples for 6–10 and 8 samples for >10 age group) milk. Amplifying the *IS1111* and the *icd* genes via nested-PCR produced a fragment of 421 bp and 556bp, respectively. Furthermore, the prevalence rate based on the *icd* gene was 7.1 % (95 % CI: 5.59%–9.08 %). Additionally, the prevalence rates based on the *IS1111* and *QpH1* genes were 142 (16.9 %) (95 % CI: 14.5–19.6) and 86 (60.6 %) (95 % CI: 52.35–68.2), respectively (Fig. 2, Fig. 3) and (Table 2, Table 3, Table 4, Table 5).

**Table 2:** Results of detecting different genes in *C. burnetii* positive milk samples using nested PCR (n = 420).

Target gene	Animal	
	Buffalo	Cattle
**IS1111**	81 (19.28 %)^a^	61 (14.52 %)^b^
**QpH1**	46 (56.79 %)^a^	40 (65.57 %)^a^
***Icd***	25 (5.95 %)^b^	35 (8/33 %)^a^

The data show the number and percentage of positive samples (n = 420). Additionally, a different letter in each row indicates significant differences at the level of p < 0.05.

**Table 3:** Results of detecting different genes in *C. burnetii* positive milk samples using nested PCR in different age groups of animals (years old).

Target gene	Animal	Age group			Total
		<6	6–10	>10	
***IS1111***	Buffalo	11^Ab^ (13.58 %)	59 ^Aa^ (72.84 %)	11 ^Ab^ (13.58 %)	81
	Cattle	9 ^Ab^ (14.75 %)	44 ^ABa^ (72.14 %)	8 ^Ab^ (13.11 %)	61
***QpH1***	Buffalo	4 ^Ab^ (8.7 %)	38 ^Ba^ (82.6 %)	4 ^ABb^ (8.7 %)	46
	Cattle	4 ^ABb^ (10 %)	30 ^BCa^ (75 %)	6 ^ABb^ (15 %)	40
***Icd***	Buffalo	2 ^Bb^ (8 %)	22 ^Ca^ (88 %)	1^Bb^ (4 %)	25
	Cattle	3 ^ABb^ (8.6 %)	30 ^Ba^ (85.7 %)	2 ^Bb^ (5.7 %)	35

∗ Different capital letters in each column (each gene separately) indicate significant differences (p < 0.05). Similarly, lowercase letters within in each row also indicate significant differences (p < 0.05).

**Table 4:** The results of detection different genes in *C. burnetii* positive milk samples by nested PCR in different season.

Target gene	Animal	Season				Total
		Spring	Summer	Autumn	Winter	
***IS1111***	Buffalo	12^Ab^ (14.8 %)	39^Aa^ (48.1 %)	30 ^Aa^ (37.1 %)	0^Ac^ (0.0 %)	81
	Cattle	7 ^ABb^ (11.5 %)	31 ^Aa^ (50.1 %)	23 ^Aa^ (38.4 %)	0 ^Ac^ (0.0 %)	61
**QpH1**	Buffalo	4 ^Bb^ (8.7 %)	23 ^Ba^ (50 %)	19 ^Ba^ (41.3 %)	0 ^Ac^ (0.0 %)	46
	Cattle	5 ^Bb^ (12.5 %)	22 ^Ba^ (55 %)	13 ^Bb^ (32.5 %)	0 ^Ac^ (0.0 %)	40
***Icd***	Buffalo	3 ^Bb^ (12 %)	12 ^Ca^ (48 %)	10 ^Ba^ (40 %)	0 ^Ac^ (0.0 %)	25
	Cattle	4 ^Bb^ (11.4 %)	17 ^ABa^ (48.6 %)	14 ^Ba^ (40 %)	0 ^Ac^ (0.0 %)	35

∗ Different capital letters in each column (each gene separately) show significant differences (p˂0.05). Also, small letters show in each raw show significant differences (p˂0.05).

**Table 5:** The results of detection different genes in *C. burnetii* positive milk samples by nested PCR in different region of the study.

Target gene	Animal	Region			Total
		North	Center	South	
***IS1111***	Buffalo	43^Aa^ (53 %)	26 ^Ab^ (32 %)	12 ^Ac^ (15 %)	81
	Cattle	32 ^Ba^ (52.5 %)	20 ^ABb^ (32.3 %)	9 ^ABc^ (15.2 %)	61
***QpH1***	Buffalo	25 ^Ca^ (54.3 %)	16 ^BCb^ (34.8 %)	5 ^BCc^ (10.9 %)	46
	Cattle	25 ^Ca^ (62.5 %)	11 ^CDb^ (27.5 %)	4 ^BCc^ (10 %)	40
***Icd***	Buffalo	15 ^Da^ (60 %)	8 ^Db^ (32 %)	2 ^Cc^ (8 %)	25
	Cattle	16 ^Da^ (45.7 %)	14 ^Cb^ (40 %)	5 ^BCc^ (14.3 %)	35

∗ Different capital letters in each column show significant differences (p˂0.05). Also, small letters show in each raw show significant differences (p˂0.05).

The results based on seasons showed that the highest prevalence of *C. burnetii* was in the summer season. Out of 219 samples, 70 samples (31.1 %) were positive, and the lowest prevalence was in the winter season where none of the 210 samples taken from cow and buffalo milk were positive. The results revealed a statistically significant difference (p < 0.05) in the disease prevalence during different seasons, and the summer season had the highest frequency of contamination by this bacterium compared to other seasons. Furthermore, the results based on geographical regions indicated that the highest contamination rate was in the northern region, where 75 out of 275 milk samples (27.3 %) were positive, and the lowest contamination rate was in the southern region, where 21 out of 284 milk samples (7.4 %) were positive. The results of this study also showed a significant difference (p < 0.05) in the prevalence of the disease in different regions, including the north, central and south of the province. Therefore, we can conclude that the geographical region has a significant effect on the incidence of *C. burnetii* infection and the elimination of the bacterium from cow and buffalo milk.

Based on animal type, out of the total 420 milk samples taken from cows, 81 samples (19.3 %) were positive, and out of 420 milk samples taken from buffalo, 61 samples (14.5 %) were positive. According to the results, no significant difference was found in the incidence of *C. burnetii* infection between cows and buffaloes. Additionally, the age group analysis revealed that the highest prevalence was in the age group above 10 years old, where 19 out of 4 milk samples (22.6 %) were positive, and the lowest prevalence was in the 4 to 6-year-old group where 59 out of 794 milk samples (9.9 %) were positive. Finally, the results showed a significant relationship (p < 0.05) between the age of cows and the incidence of *C. burnetii* infection in cow's milk in West Azerbaijan province. However, no significant relationship was observed between the age of buffalo and the incidence of this bacterium.

### 3.2 Identification of plasmid in C. burnetii

**Fig. 4:**
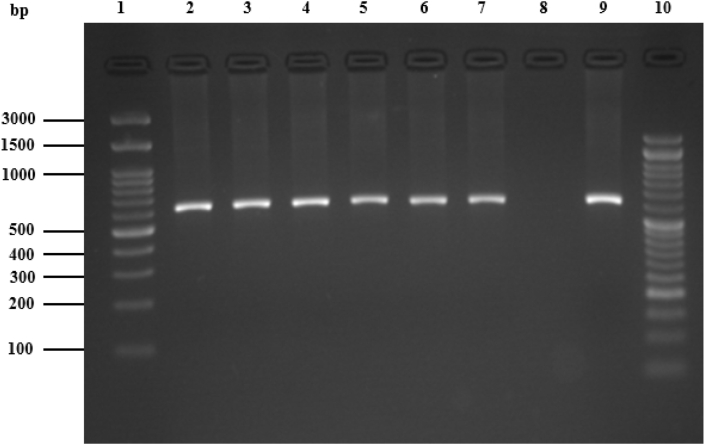
Agarose gel image of amplified fragment of *C. burnetii* cbhE*′* gene QpH1 (602bp) using nested-PCR. Lanes 1 and 10, 100-bp molecular ladder, lanes 2, 3, 4, 5, 6, 7 positive samples, lane 8, negative control, lane 9, positive control (*C. burnetii* standard Nine Mile strain RSA 493 Nine).

All of the *C. burnetii*-positive samples underwent nested-PCR analysis to identify plasmids. Using particular *QpH1* plasmid primers, the kinds of plasmids in the affirmative milk samples were directly identified. 68 (60.6 percent) of the 142 milk samples that tested positive for *C. burnetii* also tested positive for the *QpH1* plasmid (Fig. 4).

### 3.3 Phylogenetic analysis

**Fig. 5:**
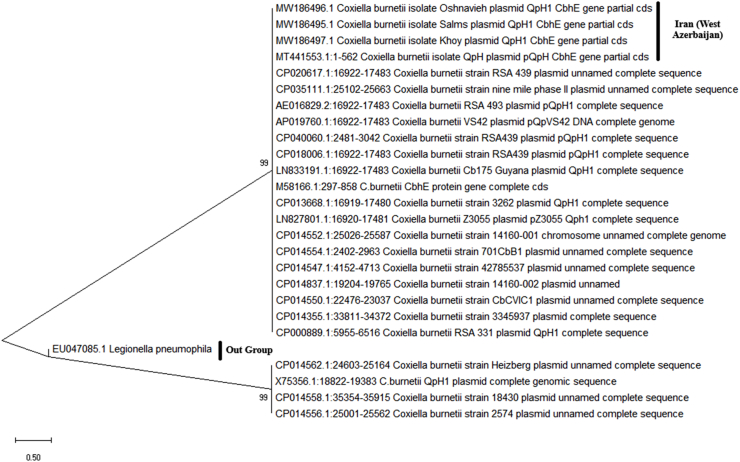
Phylogenetic inference of *C. burnetii* by (*cbhE* partial gene) sequencing.

According to a phylogenetic tree built using Maximum Likelihood method analysis of the *cbhE* partial gene, four isolates were found to be tightly grouped together and to have 99.9 % similarity, which is regarded to be the same (Fig. 5).

The nucleotide sequences of the *cbhE* gene were subjected to comparative analysis both among themselves and against sequences accessible in the NCBI gene bank through Blast analysis. The obtained sequences were then analyzed using the Mega 10 software, and a phylogenetic tree was constructed. The results showed more than 99 % similarity between the obtained sequences and the registered sequences in NCBI. Additionally, for the confirmation of the species, the sequences of four samples were determined after amplification using the *IS1111* gene and confirmed to be *C. burnetii*. After alignment and correction using the Mega 10 software, the sequences were 160 base pairs long. All four sequences showed 100 % similarity to each other, and after Blast analysis, they showed 100 % similarity with more than 50 sequences available in the gene bank from various sources and regions. Therefore, drawing a phylogenetic tree was not necessarily due to the 100 % similarity with other strains from different regions and sources.

The evolutionary history was inferred by using the Maximum Likelihood method and Tamura-Nei model [16]. The tree with the highest log likelihood (−2120.95) is shown. The percentage of trees in which the associated taxa clustered together is shown next to the branches. Initial tree(s) for the heuristic search were obtained automatically by applying Neighbor-Join and BioNJ algorithms to a matrix of pairwise distances estimated using the Maximum Composite Likelihood (MCL) approach, and then selecting the topology with superior log likelihood value. The tree is drawn to scale, with branch lengths measured in the number of substitutions per site. This analysis involved 26 nucleotide sequences. Codon positions included were 1st+2nd+3rd + Noncoding. There was a total of 562 positions in the final dataset. Evolutionary analyses were conducted in MEGA X [17].

## 4 Discussion

In the Northwest of Iran, cattle and buffalo have been demonstrated to be susceptible to the common zoonosis known as Q fever [18]. In Iran, research on Q fever in animal hosts like buffalos is uncommon, and *C. burnetii* molecular research is also uncommon. In the wake of this research, 86 (10.2 %) of the 840 milk samples examined tested positive for the *QpH1* plasmid. The bacteria are shed during parturition also in cattle, more pronounced when abortions occur. Shedding in milk is longer and can be intermittent. Buffaloes are a source of red meat for human consumption. Globally, buffalo meat accounts for 12.4 % of total beef production annually. The superior quality of buffalo meat and milk remains unknown to the general public [19,20].

In general, the *IS1111* gene was positive in 16 % of samples, while the *icd* gene was positive in 8 % of samples. Only two samples had the *IS1111* and *icd* genes found in them (ten sample of milk). This suggests that the samples examined had extremely low levels of *C. burnetii*, which needed a multicopy target to reach the sensitivity required for detection. The existence of samples that test positive just for *IS1111* is completely predicted given that *C. burnetii* strains may contain up to 110 *IS1111* elements [21].

As a result, our research is consistent with a report from a systematic review on the prevalence of *C. burnetii* in milk from various regions of Iran, which ranged from 4.22 percent to 25.55 percent [22]. Recent research in Chile [23] found that *C. burnetii* was detected in tank raw cow milk at a prevalence of 2.1 percent, which is much lower than the observed frequency in the current study. This difference may be due to geographic location. Infection rates are at their greatest in the spring and early summer. Additionally, some researches have shown that the incidence of Q fever rises significantly during lambing season, which results in the majority of cases of Q fever being recorded in the summer in several European nations [24,25]. According to the earlier research, our research found that *C. burnetii* prevalence in milk was greatest in the summer [26]. The high prevalence of *C. burnetii* can be higher in subtropical and tropical compared to that of cooler climates due to tick activity [27].

Among the milk samples that tested positive for *IS1111*, 47.8 % also exhibited positivity for the *QpH1* plasmid, this indicates that the majority of the isolates belonged to the species that causes an acute version of the illness. Five out of eight cow milk samples (62.5 %) from research by Loftis et al. included *QpH1* plasmid, producing results that were quite similar to those of the current study [28]. *QpH1* and *QpRS* sequences were found in the serum of human patients in a different study by Zhang et al. [5]. All of the sheep samples tested positive for *C. burnetii* in research by Hilbert et al. in Germany contained plasmid *QpH1* [29]. The majority of the isolates found in pregnant women in the Netherlands had *QpH1* plasmids [30]. In the study of Abu Abdullah et al. (2022), demonstrated that *C. burnetii* exhibits a high genome plasticity and that some of the genomic features are correlated to the clinical outcome, epidemiological characteristics and geographic distribution.

Our research suggests that a specific strain of *C. burnetii* that mostly carries the *QpH1* plasmid infects animals in West Azerbaijan province Iran. However, further isolates need to be analyzed to confirm this hypothesis. Few research has been done so far on the impact of age on *C. burnetii's* milk excretion. Designing herd-flock level Q fever control and eradication strategies requires knowledge of the times of the production cycle when the risk of *C. burnetii* transmission is highest and which age groups or stock classes are responsible for infection transmission [31]. This research discovered a substantial correlation between age and *C. burnetii* excretion via cow's milk. The findings of this study are in line with other publications, which indicate that age has a major role in the risk of *C. burnetii* excretion from milk in cattle [12].

The results of the current study's phylogenetic analysis of the plasmid *QpH1* of four *C. burnetii* showed that four isolates were grouped together with nearly identical nucleotide sequences, which is consistent with findings from a recently published study that found limited evolution within genomic groups in *C. burnetii* [32]. RFLP and/or the presence of plasmid DNA may be employed to distinguish *C. burnetii* isolates despite the highly conserved genome of this organism [33]. PCR is a valuable tool for early detection of *C. burnetii* in shell-vial cultures, aiding in the diagnosis of both acute and chronic infections, as well as the detection of the bacteria in clinical specimens, including heart valves [34]. Also, nowadays newer and more accurate techniques such as MLVA, SNP and MST are used for the genotyping of *C. burnetii* and diagnosis of Q fever.

The results of the study by Abou Abdallah and colleagues (2022) indicate that *C. burnetii* strains that carry *QpRS* genes cause asymptomatic primary infections. If this hypothesis is substantiated, it would be unfeasible to implement a preventative strategy against endocarditis or other persistent, focal infections in patients who have been infected with such strains. This finding is not confounded by geographical factors, as multiple *QpRS* strains have been identified in a number of countries. Consequently, based on the findings that have been corroborated by genomic and statistical analysis, it is hypothesized that strain-specific characteristics play a significant role in determining the clinical manifestations of Q fever.

Previous studies have shown a relationship between the presence of *QpH1* and *QpDV* plasmids and acute infections or abortions [30,35]. In our study, we observed that all isolates had the *QpH1* plasmids, and none of them had the *QpRS* plasmid. This is interesting because these plasmid types are not exclusively found in acute Q fever-associated strains [35,36].

Genomic analysis has provided valuable insights into the genomic dynamics of *C. burnetii*, including its evolution, host adaptation, epidemiology, and clinical profile. Newer techniques such as multi-locus variable-number tandem repeat (VNTR) analysis (MLVA) have also been suggested as sensitive and cost-effective tools for investigating molecular epidemiology [35]. In a study by Tehrani and Ownagh (2023), for example, out of 320 horse serum samples, 26 were found to be infected with *C. burnetii*, and all of them belonged to *QpH1* plasmid strains. None of the samples in this study contained the *QpRS* gene [37].

Experiments analyzing the function of both the conserved and unique plasmid sections of *C. burnetii* are crucial for comprehending the biology of this organism. All *C. burnetii* isolates were discovered to have plasmids or plasmid-homologous sequences incorporated into their chromosomes, indicating that these sequences contain important components and/or perform critical tasks for the organism. Experiments including mutagenesis and transformation may reveal the underlying roles of conserved and unique genes [38]. It was indicated that the great sensitivity of PCR necessitates its use in the diagnosis of Q fever [39].

This phylogenetic analysis study was performed using *QpH1* fragments obtained from *C. burnetii* species isolated from cow and buffalo milk. The results indicated a close evolutionary relationship with the same species reported in China, the USA, Japan, Germany, France and the Netherlands. Phylogenetic analysis of *C. burnetii* in different milk samples revealed a close evolutionary relationship with *C. burnetii* strains isolated from humans of the same or different species, suggesting that the observed association is attributable to the close interaction between infected animals and humans. This interaction is likely a significant risk factor for zoonotic infections, including Q fever in humans. The lack of surveillance for *C. burnetii* in the region highlights the urgent need to address the potential pathogenic consequences of this bacterium and its resulting diseases.

In addition, the measurable findings of PCR may be used to monitor treatment, particularly in chronic cases. Nevertheless, given the plasmid types, PCR might be used to classify the isolates. Consequently, it might be used in epidemiological investigations. Although the plasmids can be studied using the nested PCR technique, *C. burnetii* may not be detected in milk samples due to the presence of the inhibitors. In a broader sense, these strains may also include plasmids such as *QpDG*, *QpRS*, or *QpDV*. When accessible, the *C. burnetii* plasmid types in these samples may be determined using PCR using primers specific for other plasmids.

## 5 Conclusion

To the best of our knowledge, this study represents the inaugural investigation to document the identification of *C. burnetii* plasmids in milk from cattle and buffalo in Iran. Our research gives more evidence for this significant condition in Iran. Due to the severity of the flu-like symptoms, we feel our data support the notion that the prevalence of animal Q fever in Iran is grossly underestimated. To determine the prevalence of *C. burnetii*, it will be necessary to conduct more research that include acute Q fever in people and abortion in animals. In the event of temperature and time optimizations, it is important to note that the nested-PCR discovery findings may be accomplished by modifying the extraction procedure.

## CRediT authorship contribution statement

**Peyman Khademi:** Writing – review & editing, Writing – original draft, Software, Methodology, Formal analysis, Data curation. **Amir Tukmechi:** Writing – original draft. **Abdulghaffar Ownagh:** Writing – review & editing, Writing – original draft.

## Availability of data and materials

The data are available from the corresponding author upon reasonable request.

## Competing interests

The authors declare that they have no conflicts of interest. All authors have full control over the primary data and agree to allow the review of their data upon request.

## Data

All authors have full control of all primary data and they agree to allow review of their data upon request.

## Funding

The authors would like to thank the Vice-Chancellor for Research and Technology at Urmia University for funding the PostDoc project (D10/647).

## Declaration of competing interest

The authors declare that they have no conflicts of interest.
